# Mechanisms and Novel Therapeutic Approaches for Gliomas

**DOI:** 10.3390/biomedicines14081704

**Published:** 2026-07-29

**Authors:** Maria V. Chatziathanasiadou

**Affiliations:** Laboratory of Biochemistry, Faculty of Medicine, University of Thessaly, Biopolis, 41500 Larissa, Greece; mchatziath@uth.gr

Gliomas are a broad category of primary brain tumours accounting for 80% of all malignant central nervous system (CNS) tumours [1]; however, their underlying etiology remains largely unknown. The 2021 WHO classification defines their taxonomy based on clinical, histopathological, and core molecular characteristics, with the main categories being adult-type diffuse gliomas, pediatric-type diffuse low- and high-grade gliomas, ependymomas, and circumscribed astrocytic gliomas [2]. Although gliomas represent a highly heterogeneous group with grades ranging from 1 to 4, higher-grade tumours share common clinical hurdles. These include intratumoral heterogeneity, an immunosuppressive microenvironment [3], and the physiological restriction of the blood–brain barrier, which strictly limits current treatment options. For most cases, maximal safe surgical resection is the primary intervention, while adjuvant radiation and chemotherapy remain the standard follow-up options [4]. Clinical breakthroughs have substantially slowed, leaving the standard of care unadvanced for over ten years in adult diffuse gliomas [5] and for more than twenty years specifically in glioblastoma [6]. This enduring clinical plateau underscores the critical need to decipher the complex molecular mechanisms driving these malignancies and develop tools for better diagnosis and tumour characterization, thereby paving the way for novel and more suitable therapeutic strategies. Addressing these challenges is the central objective of this Special Issue.

The molecular landscape of gliomas remains largely unmapped; hence, further basic research is essential to fully characterize their cellular and spatial complexity. Glioblastoma, an adult-type diffuse glioma of the CNS WHO grade 4, is an exceptionally aggressive malignancy with a dismal 5-year survival of approximately 7% [7]. Two fundamental characteristics of glioblastoma are invasion and glioma stem cells that display profound intrinsic resistance to standard radiochemotherapy, virtually guaranteeing aggressive recurrence even after maximal safe surgical resection. Crucially, recent work by Jahan et al. published within this Special Issue demonstrates that glioblastoma cells overexpress the innate immune receptor nucleotide-binding oligomerization domain-containing protein 2 (NOD2) [8]. This activation triggers epithelial–mesenchymal transition (EMT) cascades—upregulating key drivers such as the transcription factor Snail and the mesenchymal marker Vimentin—to fuel aggressive tissue invasion. Additionally, NOD2 signaling maintains the highly treatment-resistant cancer stem cell niche, displaying strong positive correlations with the core stemness markers CD44 and CD133. Interestingly, the authors successfully inhibited invasion as well as decreased the expression of these stemness markers by knocking down NOD2. These promising findings highlight the necessity for additional research as targeting the NOD2 axis in glioblastoma might lead to a new therapeutic approach to disrupt glioblastoma invasion and recurrence.

Beyond signalling rewiring due to genetic alterations, the aggressive progression of glioblastoma is fundamentally sustained by profound metabolic reprogramming. While historical research focused almost exclusively on accelerated glucose consumption and the Warburg effect, contemporary neuro-oncology increasingly recognizes that these tumours are addicted to alternative nutrients to satisfy their biosynthetic demands and consequently their growth and expansion. Among these metabolic signatures, amino acid metabolism serves as a master target. For instance, tryptophan metabolism produces the oncometabolite kynurenine, which drives tumour cell proliferation and systemic immunosuppression by exhausting cytotoxic T cells [9]. A comprehensive review published within this Special Issue precisely dissects this aspect, detailing how amino acid deprivation strategies can fundamentally disrupt the glioblastoma microenvironment. By systematically stripping the tumour niche of essential amino acid components such as arginine, glutamine, methionine and cysteine, these therapeutic approaches target a broad spectrum of oncogenic vulnerabilities, ranging from altered DNA methylation and proliferation dynamics to immunomodulatory effects [10]. Since standard treatments routinely fail against adaptable tumour metabolism, exploiting amino acid starvation or blocking metabolic checkpoints offers promising new approaches to treat glioblastoma.

As therapeutic strategies evolve toward hitting hidden molecular and metabolic vulnerabilities, standard neuroimaging practices must adapt concurrently to provide higher diagnostic resolution and accurate longitudinal tracking. A major hurdle in glioma diagnosis and monitoring is the anatomical location of the tumour, which strictly precludes the collection of multiple tissue biopsies and necessitates the development of accurate, non-invasive diagnostic methods. Addressing this diagnostic bottleneck, a comprehensive review published within this Special Issue highlights the transformative potential of advanced radiomics and radiogenomics in adult-type diffuse gliomas [11]. By converting standard, qualitative MRI images into quantitative, high-dimensional data, radiomics serves as a non-invasive “digital biopsy” capable of decoding tumour biology. Integrating these deep computational algorithms with classical histopathology allows clinicians to better stratify patient risk (e.g., distinguish pseudoprogression from true progression), predict treatment responses (e.g., MGMT promoter methylation status), and achieve highly personalized neuro-oncological monitoring (e.g., prediction of recurrence location), representing a crucial pillar in the modern management of these malignancies.

In conclusion, breaking the decades-long therapeutic plateau in glioma management demands a concerted, multidimensional approach that bridges bench-side molecular discovery with bedside clinical precision. By uncovering novel oncogenic hubs—such as aberrant receptor signalling axes and the distinct cellular reliance on amino acid profiles like arginine, glutamine, methionine, and cysteine—researchers are successfully mapping out the precise mechanisms driving tissue invasion, cancer stemness, and local immunosuppression. The emergence of multi-omics approaches will be crucial to map these complex mechanisms longitudinally within the evolving tumour microenvironment. Concurrently, translating these biological insights into effective patient management relies on the evolution of advanced clinical practices, particularly radiomics and radiogenomics. Ultimately, this era represents a pivotal convergence of basic science, clinical insights, and advanced technologies like artificial intelligence (AI)—collectively laying the foundation for targeted, next-generation therapies to disrupt glioma progression.
